# Thanks to our peer reviewers

**DOI:** 10.1002/lrh2.70046

**Published:** 2025-10-29

The publication of Issue 4 marks the completion of Volume 9 of *Learning Health Systems*. An international, trans‐disciplinary, open access publication, the journal has advanced research and scholarship on learning health systems in partnership with our reviewers. With indexing in multiple major sources and an Impact Factor of 2.6, we have achieved a publication milestone that signals a sustainable, positive trajectory. Articles from the journal had more than 142,000 full‐text views in 2024. We continue to diversify the offerings of the journal through special issues and special collections, and through the supplemental issue published each year in collaboration with Academy Health.

We are keenly aware that these achievements would not have happened without the dedicated efforts and insightful comments of all those individuals who accepted invitations to review submitted articles. With busy schedules and full commitments, these individuals found the time and energy to contribute their expertise to our authors to help ensure that their papers met (and often exceeded) the journal's high standards for publication.

Please accept our sincere gratitude for your outstanding efforts!

Charles P. Friedman, Editor in Chief

Nancy Allee, Senior Associate Editor

Linda Novak, Managing Editor

***Learning Health Systems* Peer Reviewers**

Note: These are the reviewers for articles published in all four issues of Volume 9 and for all articles currently published and posted online in Early View through September 30, 2025. Reviewers of manuscripts that were not ultimately published are also included in this list.

Julia Adler‐Milstein (United States)

Aneel Advani (United States)

Holt Anderson (United States)

Nate Apathy (United States)

Cristina Ardura‐Garcia (Cambodia)

Amir Reza Azizian (United States)

Ross Bailie (Australia)

Rebecca Baker (United States)

Timothy Beebe (United States)

Peter Bower (United Kingdom of Great Britain and Northern Ireland)

Jeffrey Brown (United States)

Michael Bushey (United States)

Michael Cantor (United States)

Yidan Cao (United States)

Harold Collard (United States)

Marisa Conte (United States)

Theresa Cullen (United States)

Jennie David (United States)

Josie Dickerson (United Kingdom of Great Britain and Northern Ireland)

Anne Douglass (United States)

Douglas Easterling (United States)

Davide Ferrari (United Kingdom of Great Britain and Northern Ireland)

Stephan Fihn (United States)

Karen Fisher (Australia)

Allen Flynn (United States)

Tom Foley (United Kingdom of Great Britain and Northern Ireland)

Kelly Foltz‐Ramos (United States)

Rachel Forcino (United States)

Patricia Franklin (United States)

Nicholas Fusco (United States)

Melissa Garrido (United States)

Cheryl Gatto (United States)

Patricia Grady (United States)

Sarah Greene (United States)

Melissa Haendel (United States)

Allyson Hall (United States)

W. Ed Hammond (United States)

Michael Harrison (United States)

Richard Harrison (United States)

Kevin Haynes (United States)

A. Jay Holmgren (United States)

Sherita House (United States)

Clarissa Hsu (United States)

Matthew Hudnall (United States)

Matthew Hudson (United States)

Claire Zabelle Kalpakjian (United States)

Dipak Kalra (Belgium)

Kathryn Keitzman (United States)

Anjum Khurshid (United States)

Amy Kilbourne (United States)

Andrew Knighton (United States)

Joseph Kolars (United States)

Nikolas Koscielniak (United States)

Greg Koski (United States)

Tony Kuo (United States)

Zach Landis‐Lewis (United States)

Jennie Larkin (United States)

Lisa Lederer (United States)

Harold Lehmann (United States)

Lei Lu (United Kingdom of Great Britain and Northern Ireland)

Brad Malin (United States)

Jill Marsteller (United States)

David McCallie (United States)

Melvin McInnis (United States)

Frank McStay (United States)

Genevieve Melton (United States)

Matthew Menear (Canada)

Michelle Meyer (United States)

Eli Mlaver (United States)

David Mosen (United States)

Nathan Pajor (United States)

Rechelle Paranal (United States)

Allison Parsons (United States)

Philip Payne (United States)

Anna Perry (United States)

Gretchen Piatt (United States)

Leon Pretorius (South Africa)

Wayne Psek (United States)

James Ralston (United States)

Hanieh Razzaghi (United States)

Melissa Rethlefsen (United States)

Rachel Richesson (United States)

Jennifer Ridgeway (United States)

Joshua Rubin (United States)

Lauren Russell (United States)

Lucy Savitz (United States)

Gregory Sawicki (United States)

Virginia Schmied (Australia)

Julie Schmittdiel (United States)

Christine Schuler (United States)

Philip Scott (United Kingdom of Great Britain and Northern Ireland)

Michael Seid (United States)

Rachel Shapiro (United States)

Chistopher Sharp (United States)

Sina Shool (Iran)

Colin Simpson (New Zealand)

Amy Sitapati (United States)

Geoffrey Siwo (United States)

Mary Slavin (United States)

Caren Stalburg (United States)

Umberto Tachinardi (United States)

Ming Tai‐Seale (United States)

Anna Taylor (South Africa)

Jessica Tenenbaum (United States)

Mary Tobin (United States)

Susan Trinidad (United States)

Aricca Van Citters (United States)

Shelley Vanderhout (Canada)

Robert Verheij (Netherlands)

Alexandra Vinson (United States)

Jim Walker (United States)

William Weeks (United States)

Mark Weiner (United States)

Matt Whalen (United States)

La'Marcus Wingate (United States)

Brianne Wood (Canada)

Merrick Zwarenstein (Canada)

